# The Dsc complex and its role in Golgi quality control

**DOI:** 10.1042/BST20230375

**Published:** 2024-09-26

**Authors:** Yannick Weyer, David Teis

**Affiliations:** Institute of Molecular Biochemistry, Biocenter, Medical University of Innsbruck, 6020 Innsbruck, Austria

**Keywords:** Golgi, quality control, ubiquitin

## Abstract

Membrane proteins play crucial roles in cellular functions. However, processes such as the insertion of membrane proteins into the endoplasmic reticulum (ER), their folding into native structures, the assembly of multi-subunit membrane protein complexes, and their targeting from the ER to specific organelles are prone to errors and have a relatively high failure rate. To prevent the accumulation of defective or orphaned membrane proteins, quality control mechanisms assess folding, quantity, and localization of these proteins. This quality control is vital for preserving organelle integrity and maintaining cellular health. In this mini-review, we will focus on how selective membrane protein quality control at the Golgi apparatus, particularly through the defective for SREBP cleavage (Dsc) ubiquitin ligase complex, detects orphaned proteins and prevents their mis-localization to other organelles.

## Membrane protein quality control

Membrane proteins execute essential cellular functions including nutrient/ion transport, lipid metabolism and signal transduction. Therefore, the quality, the quantity and the localization of membrane proteins is monitored [1] by the molecular machineries that insert proteins co- or post-translationally into membranes [2], by the chaperones that support membrane protein folding and assembly with their cognate partner proteins [3], by the transport complexes that deliver membrane proteins to their designated organelles and [4] by ubiquitin ligases that detect, ubiquitylate and selectively degrade mis-folded membrane proteins, as well as orphaned membrane proteins (membrane proteins that fail to assemble with their cognate partners or mis-localize). Here, we will focus on how selective membrane protein degradation by the defective for SREBP cleavage (Dsc) ubiquitin ligase complex along the secretory pathway contributes to membrane protein quality control.

**Figure 1.**
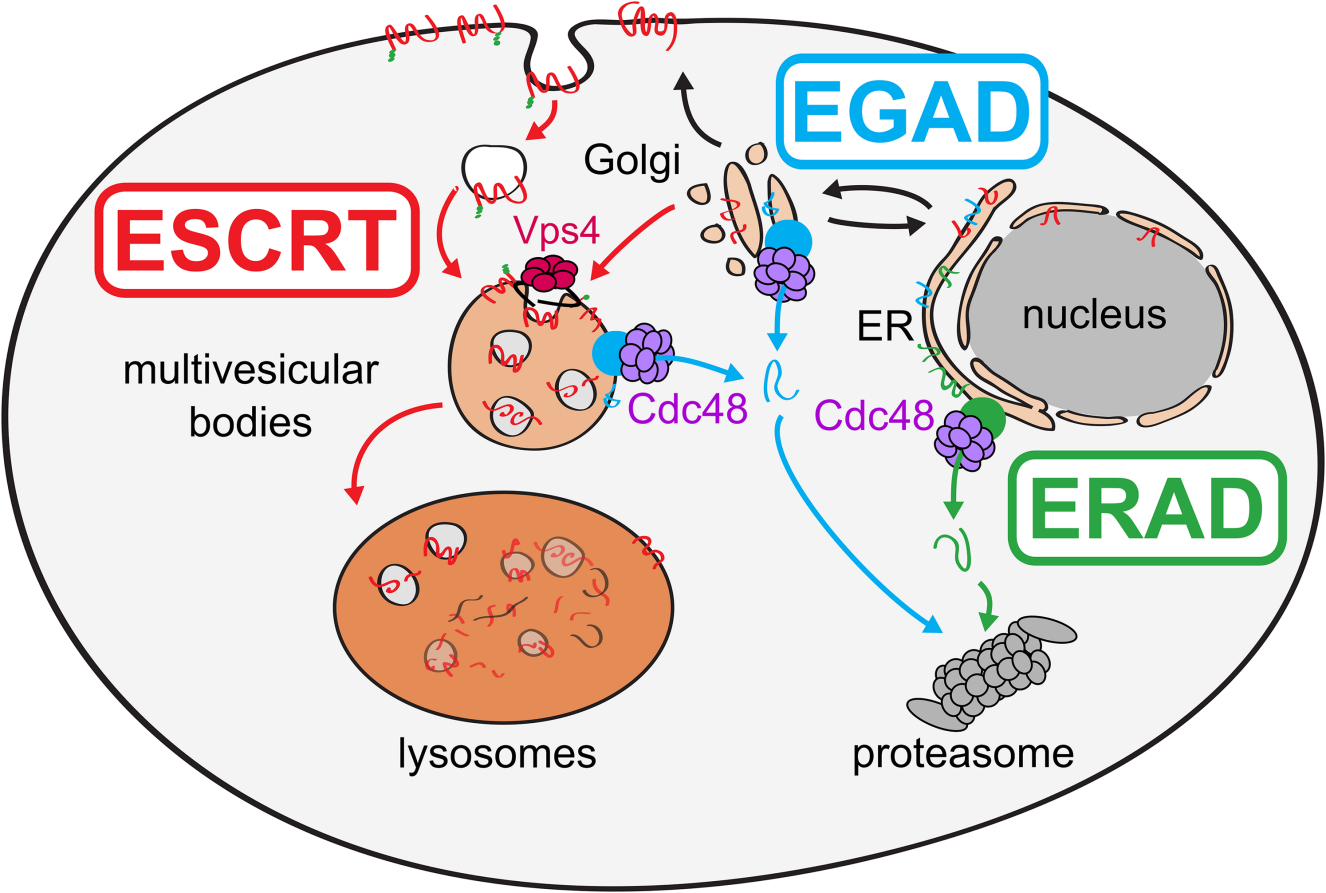
Schematic representation of ubiquitin dependent protein degradation of membrane proteins from the secretory pathway in *S. cerevisiae*. The ER associated degradation (ERAD), and the endosome and Golgi associated degradation (EGAD) use the AAA ATPase Cdc48 for membrane extraction. The endosomal sorting complexes required for transport (ESCRT) require the AAA-ATPase Vps4 for the biogenesis of multivesicular bodies (MVB). Figure is adapted from Schmidt et al. [8] licensed under Creative Commons CC BY-NC-ND 4.0.

A key step in the post-translational quality control for membrane proteins in the secretory pathway is mediated by the Endoplasmic Reticulum (ER) Associated Degradation (ERAD) machineries in the membranes of the ER and the inner nuclear membrane (INM). During ERAD, membrane proteins are recognized, ubiquitylated, extracted from the ER or INM by the mechano-enzyme Cdc48/p97/VCP and targeted to proteasomes for degradation. ERAD is probably the best understood organelle quality control system, yet some questions, including the molecular mechanism of substrate selectivity, retro-translocation, and membrane extraction are not fully understood (recently reviewed in: [1–3] (Figure 1). Once membrane proteins have cleared ER quality control and exit the ER into the Golgi, most ubiquitylated membrane proteins are targeted along the multivesicular body (MVB) pathway into the lysosome for degradation. Just how ubiquitin ligase complexes detect mis-folded and orphaned proteins in post-ER compartments is not well understood. Yet, when ubiquitylated membrane proteins localize to endosomes, they can be detected by the Endosomal Sorting Complexes Required for Transport (ESCRT). ESCRT-0, -I and -II bind directly to ubiquitylated proteins and recruit ESCRT-III and Vps4 to drive the membrane remodeling reactions required to bud cargo laden intraluminal vesicles (ILVs) into the lumen of endosomes, which leads to the formation of MVBs [4,5]. The fusion of MVBs with lysosomes leads to the degradation of ILVs and their cargo.

An additional degradation pathway that operates in the secretory pathway of budding yeast, *Saccharomyces cerevisiae*, is the Endosome and Golgi Associated proteasomal Degradation (EGAD) pathway (Figure 1) [6–10]. Central for the function of the EGAD pathway, is the Dsc ubiquitin ligase complex. In this mini-review, we will focus on the Dsc complex, and discuss how it could detect and ubiquitylate orphaned membrane proteins at the Golgi for ESCRT mediated lysosomal and EGAD mediated proteasomal degradation.

## The Dsc complex mediates PQC in post-ER compartments

The Dsc complex was discovered by Peter Espenshade and his team in *Schizosaccharomyces pombe* (fission yeast), in genetic screens for genes that were required for the proteolytic activation of the membrane bound transcription factor Sre1 (the orthologue of the mammalian SREBP). Hence the name: defective for SREBP cleavage (Dsc) complex [9].

## Functions of the Dsc complex in *S. pombe*

In *S. pombe*, the Dsc complex promotes the proteolytic activation of Sre1, together with Cdc48 and the Golgi resident rhomboid protease Rbd2 [9,11–13]. The proteolytic cleavage of Sre1 releases a soluble SREBP N-terminal transcription factor domain into the cytosol to translocate into the nucleus and regulate gene expression. This happens under conditions of low ergosterol levels or low oxygen levels [14], because then, the ER-resident Sre1 in complex with the SREBP cleavage-activating protein (Scp1, fission yeast SCAP) is transported from the ER to the Golgi. At the Golgi, Sre1 encounters the Dsc complex and the intramembrane protease Rbd2. Sre1 cleavage at the Golgi requires the E2 ubiquitin conjugating enzyme Ubc4 and the RING domain of the Dsc1 ubiquitin E3 ligase [9], along with Rbd2 mediated intramembrane cleavage of Sre1 [13,15], probably occurring in that order. The current model suggests that Dsc complex mediated ubiquitylation of Sre1 (the ubiquitylated lysine residues in Sre1 have not been identified) promotes Rbd2 mediated cleavage. Once Sre1 is cleaved, Scp1 recycles back to the ER for additional rounds of Sre1 binding and transport [16]. Defects in Sre1 cleavage cause Scp1 degradation.

On the mechanistic level, it is becoming clear that Sre1 cleavage could be co-ordinated with Cdc48 recruitment, via the SHP domain of Rbd2 [15]. In absence of functional Rbd2, Sre1 is ubiquitylated, extracted from membranes and degraded at proteasomes, which requires Dsc complex activity [15]. Hence, it seems that Rbd2 could control the balance of Sre1 activation or degradation [15]. Yet, Cdc48 and its cofactor Ufd1 are not directly required for Rbd2 mediated Sre1 cleavage [17]. Rather Cdc48-Ufd1 are required for trafficking of the Dsc complex to the Golgi, as is the RING domain of E3 ligase Dsc1 and Ubc4 (E2) [15,17,18]. Currently, it is unknown if the Dsc complex has additional substrates in *S. pombe*.

## Functions of the Dsc complex in *S. cerevisiae*

The genome of *S. cerevisiae* encodes orthologues of the Dsc complex, but it does not encode orthologues of Sre1.

Most known substrates of the Dsc complex are degraded in an ESCRT dependent manner in lysosomes/vacuoles or in an EGAD dependent manner by membrane extraction and subsequent proteasomal degradation. Dsc complex substrates, which are degraded in vacuoles include native vacuolar Zn^2+^ transporters Cot1 and Zrt3, as well as mutant proteins such as Zrt3* (a mutant version of the Zn^2+^ transporter Zrt3, which lacks the last of its eight transmembrane domains (TMDs)), over-expressed Yif1 and a mutant version of the SNARE protein Pep12, that carries a mutation in its C-terminal TMD [19–22]. Tul1 can also conjugate ubiquitin to the amine head group of phosphatidylethanolamine (PE), resulting in formation of Ub-PE on endosomes and on the vacuole/lysosome. This phospholipid ubiquitylation leads to the recruitment of the ESCRT components to liposomes *in vitro* [23]. Unbiased genetic screens from the Espenshade lab [10,12] and from us [7] revealed a negative genetic interaction between the Dsc complex and the ESCRT machinery. The negative genetic interaction indicates that the Dsc complex operates not only upstream of the ESCRT machinery, but also must mediate other important cellular functions.

Following up on this observation, we discovered that the Dsc complex is the central mediator of endosome and Golgi associated degradation (EGAD) [7]. EGAD involves the Dsc complex dependent ubiquitylation of membrane proteins, and the subsequent extraction of these ubiquitylated membrane proteins by Cdc48 for proteasomal degradation. In that sense, the mechanism of EGAD appears to be conceptually related to ERAD [1–3].

**Figure 2.**
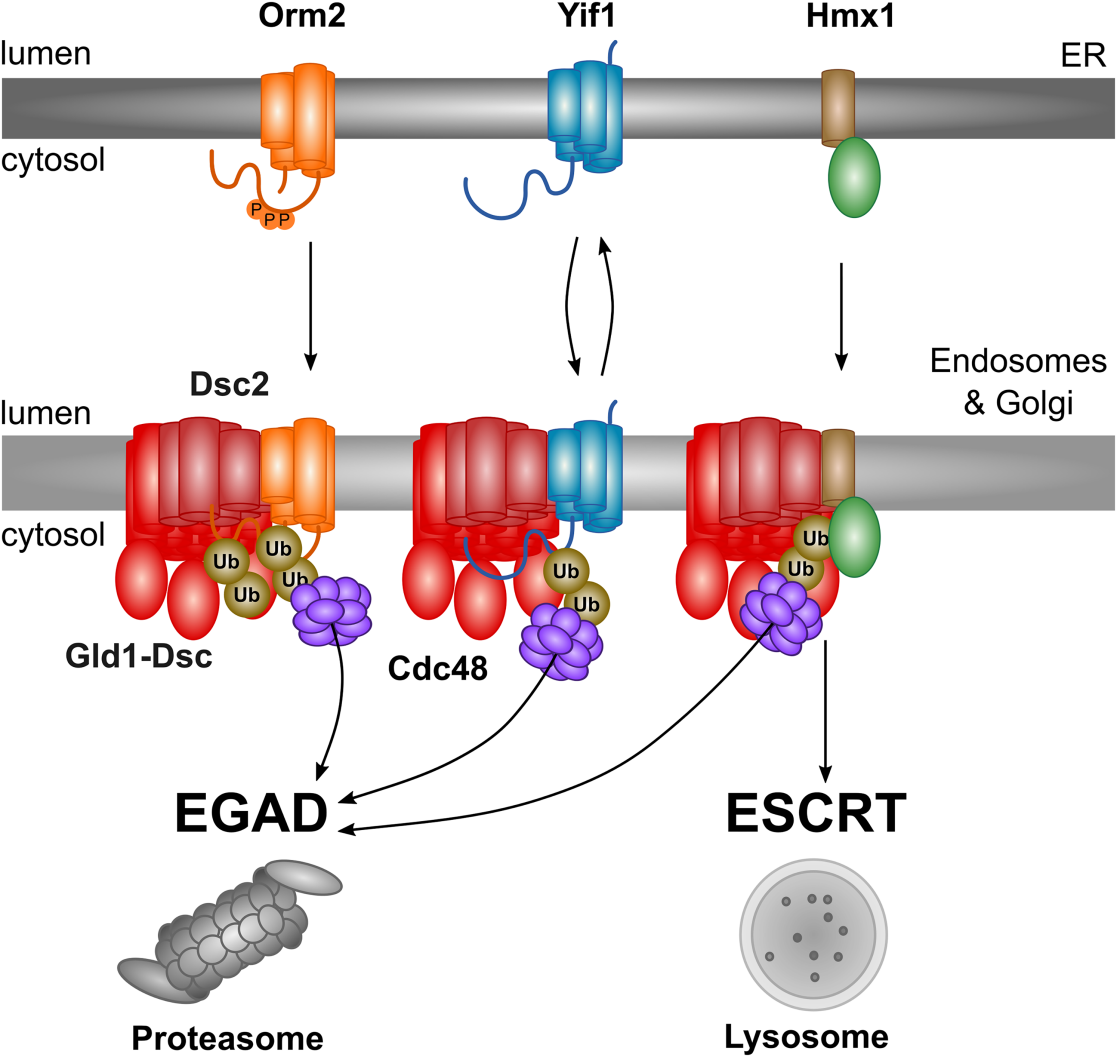
Schematic representation and summary of Dsc complex mediated degradation of transmembrane proteins. Phosphorylated Orm2 is targeted to the Golgi and endosomes where it is detected by the Dsc complex. Yif1 is a Golgi resident protein that is detected by the Dsc complex. In the case of Orm2 and Yif1, substrate detection results in poly-ubiquitylation followed by Cdc48 driven membrane extraction, which finally leads to proteasomal degradation. The ER resident heme-oxygenase Hmx1 is detected by the Dsc complex at the Golgi and endosomes; however, Hmx1 poly-ubiquitylation results either in Cdc48 dependent membrane extraction and proteasomal degradation, or in ESCRT dependent lysosomal/vacuolar degradation.

The first endogenous EGAD substrate, that we identified was Orm2 [7]. Orm2 is an ER-resident protein that downmodulates the enzymatic activity of the serine palmitoyltransferase (SPT), which catalyses the first and rate limiting step of sphingolipid (SL) synthesis [24]. The SPT is a multi-subunit protein complex that condenses palmitoyl-CoA with serine to generate 3-ketodihydrosphingosine. Orm2 and its paralogue Orm1 directly interact with the SPT and repress its activity [25–28]. The Orm2 mediated inhibition of SPT activity is homeostatically controlled by TORC2 and its downstream effector kinase Ypk1. When SL levels drop, TORC2 is activated by tensile plasma membrane stress, which results in Ypk1 activation that in turn phosphorylates Orm2 on N-terminal serine residues S46, 47, 48 [29,30]. Phosphorylated Orm2 no longer efficiently interacts with the SPT, and is targeted to the Golgi in a COPII dependent manner [7]. Orm2 export from the ER involves the rhomboid pseudo-protease Dfm1, which somehow chaperones Orm2 for COPII mediated ER export into the Golgi [31]. At the Golgi, Orm2 interacts with the Dsc complex leading to its Tul1 dependent ubiquitylation, followed by Cdc48 mediated membrane extraction and degradation at cytosolic proteasomes. In addition to Orm2, we have identified additional substrates of the Dsc complex including the heme oxygenase Hmx1 and Yif1 [6]. Some Dsc substrates will be exclusively extracted by Cdc48 for EGAD (Orm2 and Yif1), while other Dsc substrates (e.g. Hmx1) will undergo EGAD dependent proteasomal degradation or ESCRT dependent vacuolar degradation [6] (Figure 2).

To detect these substrates, the Dsc complex uses its rhomboid pseudo-protease subunit, Dsc2 [6]. We speculate that Dsc2 can thin the lipid bilayer and thereby assess the hydrophobic length of α-helical TMDs of proteins at the Golgi [6]. Thereby, the Dsc complex interacts with orphaned ER (Orm2, Hmx1) and Golgi proteins (Yif1) that have shorter TMDs and ubiquitylates them for targeted degradation. Hence, in budding yeast, the Dsc complex mediates protein quality control by the selective degradation of orphaned proteins at the Golgi and prevents the spreading of orphaned proteins across other organelles.

## Molecular architecture of the Dsc complex

The *S. pombe* Dsc complex consists of five predicted transmembrane subunits (Dsc1-5) that work together with the AAA-ATPase Cdc48 to induce proteolytic processing of Sre1 [9,11,12]. All components, except for Dsc4 (which likely corresponds to the budding yeast trafficking adaptors Gld1 or Vld1, as discussed further below), are conserved in *S. cerevisiae* [10,32] and orthologues of the Dsc complex components can be found in plants (Dsc1-3,5) and mammalian cells (Dsc2,3,5) [7,11].

**Figure 3.**
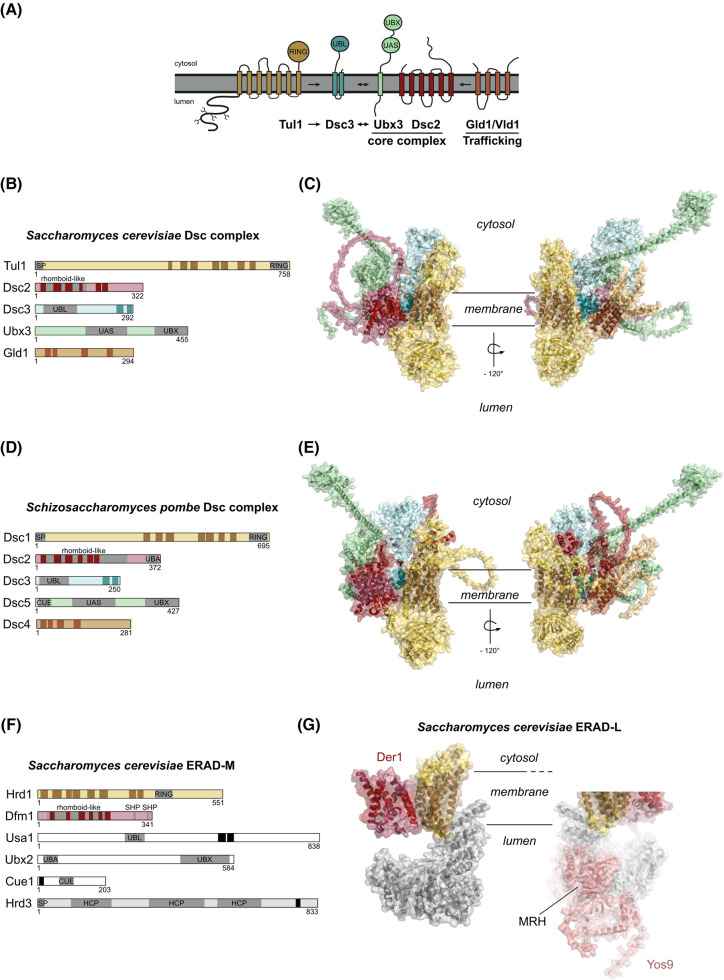
Models of the *Saccharomyces cerevisiae* (A, B) and *Schizosaccharomyces pombe* (D) Dsc complex members. Tul1 contains a N-terminal signal peptide (SP) sequence and a luminal domain with multiple glycosylation sites, seven predicted transmembrane domains and a C-terminal cytosolic RING domain. Rhomboid-like folds in Dsc2 are indicated with gray boxes. Dsc2 for *S. pombe* contains a C-terminal ubiquitin-associated (UBA) domain. Dsc3 contains an N-terminal ubiquitin-like (UBL). Ubx3 and Dsc5 contain an internal ubiquitin-associating (UAS) domain of unknown function and a C-terminal UBX domain that is known to interact with the AAA-ATPase Cdc48. Dsc5 contains an N-terminal coupling of ubiquitin to ER degradation (CUE) domain. Transmembrane domains (TMDs) are indicated with dark boxes and were predicted with DeepTMHMM. (**A**) Figure is adapted from Schmidt et al. [8]. (**C, E**) AlphaFold2 predictions of the indicated complexes and their members: Dsc1/Tul1 (gold), Dsc2 (red), Dsc3 (blue), Dsc4/Gld1 (brown), and Dsc5/Ubx3 (green). Resulting files were visualized with PyMOL, TMDs were predicted with DeepTMHMM and colored in dark. Left panel shows front view, right panel is rotated −120° around the *y*-axis. (**F**) Domains are indicated as in (**B, D**). Hrd3 contains three Haemolysin coregulated protein-like (HCP) domains. Crystal structure of *Saccharomyces cerevisiae* ERAD-L complexes. Left panel shows Der1 (red), Hrd1 (gold), and Hrd3 (gray) from PDB (6vjz). Right panel additionally shows Yos9 (pink) from PDB (6vk3). Files were visualized with PyMOL, TMDs were predicted with DeepTMHMM and colored in dark [81].

We have used AlphaFold2 (AF2) [33] to predict the structure of the *S. cerevisiae* Gld1-Dsc *complex* and *S. pombe* Dsc complex (Figure 3C,E). AF2 returned predicted models with relatively high overall PMT (0,65) and iPMT (0,65) values. The predicted subunit architecture is consistent with previous biochemical experiments [6,11,32]. As expected, the overall domain and subunit architecture of the Dsc complex of *S. pombe* and *S. cerevisiae* was similar (Figure 3A–E). The E3 ubiquitin ligase (Tul1/Dsc1) is linked via Dsc3 to the core complex which consists of the rhomboid pseudo-protease Dsc2, the Cdc48 recruitment factor Ubx3 (Dsc5), and a trafficking adaptor (Gld1, Vld1/Dsc4) [11,32].

## Tul1 — the E3 ubiquitin ligase, co-operates with the E2 enzyme Ubc4

Tul1 (transmembrane ubiquitin ligase 1) (Dsc1) is the transmembrane ubiquitin E3 ligase with a large glycosylated luminal N-terminus, 7 predicted TMDs, and a C-terminal RING-H2 domain (Figure 3A–E). AF2 models the luminal N-terminus into a barrel-like fold (Figure 3C,E), with distant similarities to MRH (Mannose 6-phosphate receptor homology) domains, like the MRH domain of the ERAD-L factor Yos9, which associates with the ubiquitin E3 ligase Hrd1/Hrd3 during ERAD-L (Figure 3G) [34].

To orchestrate the ubiquitylation of substrates Tul1 associates via its RING domain with the E2 enzyme Ubc4 [18]. Ubc4 can conjugate mono-, oligo-, and K48 or K63 poly-ubiquitin chains together with HECT and RING type ligases [35,36]. In budding yeast, Ubc4 is paralogous to Ubc5, which is preferentially up-regulated during stress conditions [37,38]. Since the Dsc complex can conjugate K48 and K63 poly-ubiquitin chains [6], we speculate that the formation of the different poly-ubiquitin chains might depend on how the Dsc complex interacts with its substrates. Yet, Tul1 is not essential for substrate interaction. Substrate detection and substrate recruitment to the Dsc complex requires Dsc2 (see also below). Substrates with shorter TMDs may be better hydrophobically matched with Dsc2, which could increase the dwell time and, thus, successful Cdc48 engagement and membrane extraction, while faster dissociation from Dsc2 could result in the sorting of the ubiquitylated substrates into the ESCRT pathway [6]. We propose that Tul1 and Ubc4 set a priming mono- or oligo-ubiquitination, that can be extended and/or branched into different multi- or poly-ubiquitin chains. For EGAD, it is likely that the conjugated ubiquitin residues are elongated into K48 poly-ubiquitin chains. Cdc48 could bring along Ufd2 as an E4 ubiquitin ligase to extend the priming ubiquitination mediated by Ubc4-Tul1 into a K48 poly-ubiquitin chain. This would reinforce Cdc48 mediated membrane extraction or subsequent proteasomal degradation or both [39–43]. Orm2 and endogenous Yif1 follow this pathway [6].

For ESCRT mediated vacuolar degradation, ubiquitylated residues are either not elongated or elongated into K63 poly-ubiquitin chains. Elongation could involve other E3 ubiquitin ligases such as Rsp5 or Pib1 [44,45].

Ubiquitylation is often antagonized or edited by deubiquitylation (DUBs) enzymes. Therefore, DUBs contribute to the specificity of the ubiquitin code, and remove, trim or rearrange the ubiquitin chains and their linkage [46]. So far, no DUBs have been identified to function together with the Dsc complex.

Tul1 is evolutionarily conserved among a wide range of fungi and plants. In *Arabidopsis thaliana*, Tul1 has two orthologues, Fly1 and Fly2, which function redundantly in establishing seed coat mucilage. Fly1 and Fly2 co-localize with both Golgi and late endosomal markers, where both control pectin methylesterifications presumably by ubiquitylating proteins involved in mucilage carbohydrate modification [47,48]. Tul1 is not conserved in humans and other mammals. Yet, the human genome has an 8–10 fold expanded repertoire of ubiquitin ligases, including several polytopic transmembrane RING domain E3 ligases at the Golgi, such as MARCH1, MARCH4, and MARCH9 [49,50]. Given that Tul1 is not essential for substrate recognition, it is likely that other ubiquitin ligases contribute to protein quality control at the Golgi in human cells.

## Dsc3 recruits Tul1 to the core complex

Based on biochemical experiments Dsc3 is required to recruit Tul1 to the Dsc complex [6,10,32]. Consistently, AF2 models suggest Dsc3 is wedged between Tul1 and Dsc2 and thereby links Tul1 to the rhomboid pseudo-protease Dsc2 and to the Cdc48 adaptor Ubx3. Little is known about Dsc3, except that it contains an N-terminal UBL domain and two predicted TMDs at its C-terminus [10] (Figure 3B–E). In yeast, UBL domain containing proteins have been reported to recruit the 26S proteasome; however, the exact function of these domains in protein degradation remains elusive [51]. With an N-terminal UBL fold, the domain architecture of Dsc3 is reminiscent to that of the ERAD associated protein Usa1 (Figure 3A–F). Interestingly, both Dsc3 (EGAD) and Usa1 (ERAD) seem to connect their respective ubiquitin E3 ligases to the other subunits of the respective machineries [10,52].

Dsc3 shows remote homology to the transmembrane and ubiquitin-like domain-containing protein 1/2 (TMUB1/2) protein family, which also contain an UBL fold and two C-terminal TMDs [7]. TMUB1 can associate with the mammalian RNF185, and TMEM259/Membralin [53] or gp78 ERAD ubiquitin ligase complexes for protein quality control and the regulation of degradation and ubiquitylation process of the HMG-CoA reductase [54]. In these processes, TMUB1 might function as an escortase for substrate retrotranslocation by safeguarding the movement of the substrate TMD from the membrane to p97/VCP [55]. During this process, the UBL domain of TMUB1 is proposed to directly recruit the mechano-enzyme p97/VCP.

## The Dsc core complex: Ubx3-Dsc2-Gld1/Vld1

In budding yeast, Ubx3 and Dsc2 form a subcomplex that interacts with one of two trafficking adaptors, Gld1 or Vld1 [10,32]. Since Dsc2 and one of the two trafficking adaptors are required to export Ubx3 from the ER (and vice versa), it is likely that the assembly of the Ubx3-Dsc2 core complex occurs at the ER [32]. Likewise, Ubx3/Dsc5 and Dsc2 form a subcomplex that interacts with Dsc4 in *S. pombe* [11].

## Ubx3 — the Cdc48/VCP recruitment factor

Ubx3 (Dsc5), belongs to the family of ubiquitin regulatory X (UBX domain) containing proteins. This family is evolutionarily conserved and recruits Cdc48/VCP/p97 with their UBX domain [12,56–58]. Ubx3 has a conceptual ERAD counterpart, namely Ubx2, and it is related to FAS-associated factor 2 (FAF2 or UBXD8 in mammals) and contains a thioredoxin-like UAS domain (that has no assigned function so far). In analogy of Ubx3 binding to Dsc2, UBXD8 associates with UBAC2 (an orthologue of Dsc2). This interaction retains UBXD8 at the ER and prevents its trafficking to lipid droplets [59]. UBXD8 has been reported to polymerize upon interaction with long-chain unsaturated fatty acids depending on its UAS domain *in vitro* [60]. Whether Ubx3 or UBXD8 form oligomers *in vivo* and if the UAS domain is involved in this process remains unknown. Furthermore, UBXD8 has been reported to insert into membrane with a hairpin, exposing both, its N- and C-terminus to the cytosol [61].

The fission yeast orthologue of Ubx3, Dsc5, contains a CUE domain at its N-terminus (Figure 3D), that is not conserved in budding yeast. If the CUE domain of Dsc5 is involved in the recruitment of an E2 enzyme, like it is the case for Cue1 in ERAD, is so far not clear. Studies from the Espenshade lab predict that Dsc5 and Ubx3 span the membrane once [10,12]. DeepTMHMM predictions [62] even failed to predict TMDs in Ubx3 (Figure 3B,D,F), or its ERAD counterpart Ubx2. However, due to the presence of an N-terminally located CUE domain in Dsc5/Ucp10, and the fact that its human paralogue UBXD8 inserts as a hairpin into the membrane, it is not unlikely that Ubx3 and Dsc5 could adapt a similar hairpin topology. This idea is supported by the AF2 model (Figure 3C,E).

## The trafficking adaptors Gld1 and Vld1

To reach post-ER compartments, the Dsc complex associates with two distinct trafficking adaptors in budding yeast. Gld1 (Golgi Localized Dsc complex) guides the Dsc complex to the Golgi and endosomes via the VPS (Vacuolar Protein Sorting) pathway. Vld1 (Vacuole Localized Dsc complex) transports the Dsc complex from the Golgi to the vacuolar membrane via the adaptor protein 3 (AP-3) pathway [32]. AP-3 sorting is achieved by an acidic di-leucine targeting motif at the C-terminus of Vld1. Gld1 and Vld1 span the membrane four times and compete with each other in binding of the Dsc complex to determine the subcellular localization. The interaction of the Dsc complex with either Gld1 and Vld1 is essential to mediate the trafficking of the Dsc complex out of the ER [32]. Gld1 and Vld1 are not obviously conserved in fission yeast; however, overexpression of fission yeast (*S. pombe*) Dsc4 in budding yeast (*S. cerevisiae*) can partially compensate for the loss of Gld1 and Vld1 and mediate ER exit of the Dsc complex [32]. AF2 predictions of *S. cerevisiae* Gld1-Dsc and *S. pombe* Dsc4-Dsc complexes show that Gld1 and Dsc4 could bind within a similar region of the Dsc complex (Figure 3C,E). If Gld1 and Vld1 have functions that go beyond Dsc complex trafficking is currently unclear.

## The Dsc2 rhomboid pseudo-protease

**Figure 4.**
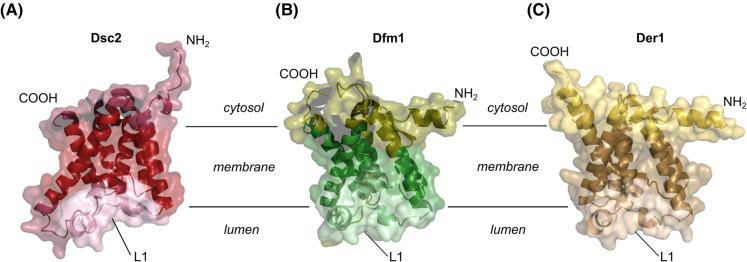
Rhomboid pseudo-proteases contain characteristic rhomboid-like folds. (**A–C**) AlphaFold predictions of the indicated proteins were visualized with PyMOL. Transmembrane domains (TMDs) are colored in dark, amino-terminus (NH_2_), carboxy-terminus (COOH), and the loop region (L1) between TMD1 and TMD2 are indicated. (**A**) Dsc2 (AF-Q08232-F1). (**B**) Dfm1 (AF- Q12743-F1). (**C**) Der1 (AF-P38307-F1).

Dsc2 is a rhomboid-like pseudo-protease and essential for substrate detection of the Dsc complex [6]. Dsc2 shares structural similarity with the Derlin family members, Dfm1 and Der1 (Figure 4A–C), and with the human ubiquitin-associated domain-containing protein 2 (UBAC2) [7,11]. Derlins and UBAC2 are implicated in ERAD processes [63–65]. In mice, UBAC2 associates together with the ERAD E3 ubiquitin ligase gp78 and the putative substrate recruitment factor LMBR1L, where they regulate Wnt/β-catenin during lymphopoiesis [66]. Genome-wide association studies suggest that polymorphisms in UBAC2 are linked to Behçet's disease, an auto-inflammatory systemic vasculitis [67–69].

In fission yeast, Dsc2 contains an ubiquitin associating (UBA) domain (Figure 3D), which can bind K63-linked poly-ubiquitin chains *in vitro* [11]. The UBA domain of Dsc2 is not required for Sre1 cleavage, suggesting that the Dsc complex may have additional cellular functions in *S. pombe* [11]. In budding yeast, Dsc2 lacks the UBA domain and so far, it is not known whether other UBA domain containing proteins associate with the Dsc complex in this organism.

Rhomboid-like pseudo-proteases lack the catalytic dyad (H-S) of the rhomboid intramembrane proteases. Yet, they retained the ability to selectively recognize TMDs of substrate proteins as well as membrane thinning activity [1,70]. *In vitro* studies and molecular dynamic simulations established that the active rhomboid protease GlpG from *Escherichia coli* can perturb the lipid bilayer to induce membrane thinning [71–74]. The loop region 1 (L1), which is composed of two short amphipathic helices situated between the TMD 1 and 2, plays an important functional role in the thinning and recognition process of GlpG [73,75]. The TMD2 and TMD5 of GlpG form a lateral gate that allows the substrate to enter the hydrophilic core of GlpG [76–79]. Like their active counterpart GlpG, members of the Derlin family (Der1, Dfm1 and Derlin1), and Dsc2 are implicated in processes that perturb and thin the lipid bilayer [6,80,81]. Dfm1, Der1 and Dsc2 contain two (or three) amphipathic helices in their L1 region that could likely play a role in membrane thinning (Figure 4A–C). We speculate that the capacity of Dsc2 to thin the lipid bilayer could promote hydrophobic matching with the shorter TMDs of Dsc complex substrates. In addition, Dsc2 might help to destabilize TMDs to overcome the energetic barrier during retro-translocation and Cdc48 mediated membrane extraction [82–84].

We conclude that the subunit architecture of the Dsc complex may be ideally geared for protein quality control at the Golgi. There, the Dsc complex could integrate lipid bilayer thickness with TMD length. Such hydrophobic TMD matching could provide general means of PQC machineries to detect and degrade orphaned protein, and limit their spreading across the cell.

## Perspectives

- It is becoming clear that the Dsc complex is central to Golgi protein quality control.
- Given that the Golgi is the central protein sorting station of the cells, we expect important contributions to membrane proteostasis and lipid homeostasis in general, by mechanistic and physiological insights into Dsc complex mediated Golgi quality control.
- In the future, several challenges need to be overcome to understand how the Dsc complex functions on the mechanistic level. This includes a detailed characterization of the mechanisms of substrate detection and substrate processing for either ESCRT or EGAD mediated degradation. Such advances could be made by resolving the structure of the Dsc complex with cryo-EM-based structural analyses and *in vitro* reconstitutions for Dsc2 mediated substrate recognition.

## Abbreviations

AP-3: adaptor protein 3
DUB: deubiquitylation
EGAD: Endosome and Golgi Associated proteasomal Degradation
ER: endoplasmic reticulum
ESCRT: Endosomal Sorting Complexes Required for Transport
ILV: intraluminal vesicle
INM: inner nuclear membrane
MVB: multivesicular body
PE: phosphatidylethanolamine
SL: sphingolipid
SPT: serine palmitoyltransferase
TMD: transmembrane domain
